# Interhemispheric claustral circuits coordinate sensory and motor cortical areas that regulate exploratory behaviors

**DOI:** 10.3389/fnsys.2014.00093

**Published:** 2014-05-19

**Authors:** Jared B. Smith, Kevin D. Alloway

**Affiliations:** ^1^Department of Engineering Science and Mechanics, Penn State UniversityUniversity Park, PA, USA; ^2^Center for Neural Engineering, Penn State UniversityUniversity Park, PA, USA; ^3^Department of Neural and Behavioral Sciences, Penn State UniversityHershey, PA, USA

**Keywords:** motor cortex, neuronal tracing, sensorimotor, visuomotor, frontal eye fields, somatosensory cortex, claustrum, visual cortex

## Abstract

The claustrum has a role in the interhemispheric transfer of certain types of sensorimotor information. Whereas the whisker region in rat motor (M1) cortex sends dense projections to the contralateral claustrum, the M1 forelimb representation does not. The claustrum sends strong ipsilateral projections to the whisker regions in M1 and somatosensory (S1) cortex, but its projections to the forelimb cortical areas are weak. These distinctions suggest that one function of the M1 projections to the contralateral claustrum is to coordinate the cortical areas that regulate peripheral sensor movements during behaviors that depend on bilateral sensory acquisition. If this hypothesis is true, then similar interhemispheric circuits should interconnect the frontal eye fields (FEF) with the contralateral claustrum and its network of projections to vision-related cortical areas. To test this hypothesis, anterograde and retrograde tracers were placed in physiologically-defined parts of the FEF and primary visual cortex (V1) in rats. We observed dense FEF projections to the contralateral claustrum that terminated in the midst of claustral neurons that project to both FEF and V1. While the FEF inputs to the claustrum come predominantly from the contralateral hemisphere, the claustral projections to FEF and V1 are primarily ipsilateral. Detailed comparison of the present results with our previous studies on somatomotor claustral circuitry revealed a well-defined functional topography in which the ventral claustrum is connected with visuomotor cortical areas and the dorsal regions are connected with somatomotor areas. These results suggest that subregions within the claustrum play a critical role in coordinating the cortical areas that regulate the acquisition of modality-specific sensory information during exploration and other behaviors that require sensory attention.

## Introduction

The claustrum is present in nearly all mammalian lineages (Kowianski et al., 1999), but its behavioral functions have not been elucidated because of its unusual geometry. Relatively narrow with a long rostrocaudal extent, the claustrum is difficult to study with standard lesion or recording techniques in a behavioral paradigm. Neuronal tracing techniques, however, have revealed many aspects of claustral circuitry, and most views about claustral functions are based on its cortical connectivity (Edelstein and Denaro, 2004; Crick and Koch, 2005; Smythies et al., 2012), which include several unique interhemispheric projections (Minciacchi et al., 1985; Li et al., 1986; Sloniewski et al., 1986a; Sadowski et al., 1997).

Using physiology-based tracing techniques in rats, we recently reported that the M1-Wh region projects strongly to the contralateral claustrum, but only weakly to the ipsilateral claustrum (Alloway et al., 2009; Colechio and Alloway, 2009; Smith and Alloway, 2010; Smith et al., 2012b). While the M1-Wh region does not receive reciprocal feedback projections from the contralateral claustrum, it is strongly innervated by the ipsilateral claustrum. By contrast, claustral connections with the M1 forelimb regions are comparatively sparse and are exclusively ipsilateral. In addition, the whisker region in S1 barrel cortex is innervated by the ipsilateral claustrum even though S1 cortex does not project to the claustrum in either hemisphere.

These findings are significant because exploratory whisking is an active sensory process that requires attention and is bilaterally-coordinated for the purpose of acquiring tactile information about the spatial features of the local environment (Towal and Hartmann, 2006; Mitchinson et al., 2007). By comparison, rodent forelimb movements are rarely if ever used used to perceive the spatial features of three-dimensional space, but are mainly concerned with supporting and moving the body through space.

The discovery of an interhemispheric claustrum-based pathway that connects the cortical regions that process whisker-related information prompted us to hypothesize that the claustrum should have similar circuit connections with the visual system. Like whisking behavior, exploratory eye movements require attention and are concerned with actively acquiring visual information to perceive a broad spatial region (Chelazzi et al., 1989; Andrews and Coppola, 1999; Wallace et al., 2013). In rats the claustrum receives a few projections from visual area 18b, but virtually none from area 17 (Miller and Vogt, 1984a; Carey and Neal, 1985). The ventral part of the rat claustrum projects to visual cortex (Li et al., 1986; Sadowski et al., 1997), but whether the claustrum has afferent or efferent connections with the FEF remains unknown. Indeed, no data indicate whether the rat claustrum is part of a disynaptic interhemispheric circuit that could coordinate the FEF and V1 areas.

Therefore, to test this hypothesis, we injected anterograde and retrograde tracers into physiologically-defined sites in FEF and V1. We compared the results, along with unreported data from our previous rat study (Smith and Alloway, 2010), to tracing data accessible from the Allen Mouse Brain Connectivity Atlas. Our findings indicate that the claustrum is part of an interhemispheric circuit that enables the FEF in one hemisphere to transmit the same information to the V1 and FEF cortical areas in the other hemisphere.

## Materials and methods

Anatomical tracing experiments were performed on three adult male Sprague-Dawley rats (Charles River) weighing 300–350 g. All procedures conformed to National Institute of Health standards and were approved by Penn State University's Institutional Animal Care and Use Committee.

### Animal surgery

Rats were initially anesthetized via intramuscular (IM) injection of a mixed solution of ketamine HCl (40 mg/kg) and xylazine (12 mg/kg). Additional IM injections of atropine methyl nitrate (0.5 mg/kg) to limit bronchial secretions, dexamethasone sodium phosphate (5 mg/kg) to reduce brain swelling, and enrofloxacin (2.5 mg/kg) to prevent infection were given before intubating the trachea through the oral cavity and ventilating the rat with oxygen. After placing the animal in a stereotaxic instrument, its heart rate, respiratory rate, end-tidal carbon dioxide, and blood oxygen were monitored (Surgivet) throughout the experimental procedure. Body temperature was regulated by a rectal probe attached to a homeothermic blanket placed on the dorsal side of the animal; a hot water blanket was placed underneath the rat as well. Ophthalmic ointment was applied to prevent corneal drying. After injecting bupivicaine into the scalp, a midline incision was performed to visualize the cranium, and a ground screw was inserted into a craniotomy over the cerebellum. Craniotomies were also made over motor cortex (1–3 mm rostral, 0.5–3 mm lateral to bregma) and visual cortex (5–7 mm caudal, 3–5 mm lateral to bregma) in both hemispheres according to coordinates in Paxinos and Watson (2007).

### Intracranial microstimulation

Intracranial microstimulation (ICMS) was done in rats to map motor cortex. Microstimulation was performed under ketamine-xylazine anesthesia to produce forepaw, whisker, or eye movements. Following microstimulation mapping, the anesthetic state was maintained with ~1% isoflurane.

Cortical stimulation was administered by ~1 MΩ saline-filled glass pipettes. Both short (80-ms, 250 Hz) and long (1-s, 100 Hz) pulse trains were administered. A biphasic constant current source (Bak Electronics, BSI-2) was used to test current levels of 10–250 μA to identify the lowest threshold at each site capable of eliciting a movement. Stimulation was conducted at multiple sites in each animal so that tracer injections could be centralized within the target region to avoid tracer leakage into surrounding representations.

The stereotaxic coordinates that evoked movements were similar to previous reports (Hall and Lindholm, 1974; Neafsey et al., 1986; Hoffer et al., 2003; Brecht et al., 2004; Haiss and Schwarz, 2005). Electrodes were positioned orthogonal to the pial surface and inserted to depths (~1 mm) that correspond to layer V, which contains corticobulbar and corticospinal neurons. The electrode was initially placed 2–3 mm lateral to the midline to identify the forepaw representation (M1-Fp). More medial sites (1–2 mm lateral) evoked brief whisker retractions (M1-Re) during 80-ms stimulation trains. At the most medial coordinates (~1 mm lateral), the electrode was advanced deeper to determine the motor representations in the medial bank of frontal cortex. At sites located 1.5–3.0 mm rostral, stimulation at depths 1.5–2.5 mm below the pial surface evoked eye movements visible to the naked eye. Further caudally, 1-s long train stimulation evoked repetitive rhythmic whisker movements at M1 (M1-RW) sites located 0.5–1.7 mm rostral to bregma. Whisker movements at M1-RW sites were frequently bilateral (Haiss and Schwarz, 2005). Both FEF and M1-RW are located deep in the medial bank of frontal cortex, but they have distinct domains along the rostral and caudal axis.

### Extracellular neuronal recordings

To identify sites in primary visual cortex (V1), the same electrodes used for ICMS mapping were used to map visual cortex. After disconnecting the electrode from the constant current source, it was connected to the headstage of a Dagan amplifier (Model 2200) so that extracellular discharges could be amplified, bandpass filtered (300–3000 Hz), and monitored with an oscilloscope and acoustic speaker. Electrodes were placed at stereotaxic coordinates (5.0–7.0 mm caudal to bregma, 2.0–4.0 mm lateral) that correspond to V1 (Paxinos and Watson, 2007), and were advanced ~400 μm into the brain to reach layer IV. Neuronal responses to visual stimulation were tested by manipulating a handheld blue LED in different directions over the ipsilateral and contralateral eyes to identify responsive areas corresponding to the monocular or binocular regions of V1. Because this procedure may not distinguish V1 from adjacent visual areas, injection sites in V1 were verified by cytoarchitectonic criteria (see Results).

### Tracer injections

Tracers were injected either iontophoretically or by pressure. For anterograde tracing, 15% solutions of FluoroRuby (FR; D-1817, Invitrogen) or biotinylated dextran amine (BDA; D-7135, Invitrogen) in 0.01 M phosphate buffered saline (PBS) were used. For retrograde tracing, 2% solutions of True Blue chloride (TB; T-1323, Invitrogen) or Fluorogold (FG; H-22845, Fluoro-Chrome) were used.

The FEF received iontophoretic injections of BDA or FG from glass pipettes (~30 μm tip). A retention current (-7.0 μA) was used to limit tracer leakage while advancing the pipette to its injection depth, where the retention current was turned off and positive current pulses of 2–5 μA (7 s on/off duty cycle) were applied for 10–20 min to eject the tracer at two depths separated by 300 μm. In one rat, a mixture of FG and BDA was iontophoretically ejected in FEF. Visual cortex received pressure injections of FR or TB from Hamilton syringes in which glass pipettes (~50 μm diameter tips) were cemented on the end of the needle. A summary of the tracer injections is in Table 1.

**Table 1:** **Summary of tracer injections from current study and previously published data (Smith and Alloway, 2010)**.

**Case**	**Left hemisphere**		**Right hemisphere**	
	**Motor region**	**Sensory region**	**Motor region**	**Sensory region**
TI-14	–	V1 (FR)	FEF (FG/BDA)	V1 (TB)
TI-15	FEF (BDA)	V1 (FR)	FEF (FG)	V1 (TB)
TI-16	FEF (BDA)	V1 (FR)	FEF (FG)	V1 (TB)
CL-01	M1-Re (FR)	–	M1-Re (FG)	–
CL-02	M1-Re (FR)	–	M1-Re (FG)	–
CL-03	M1-Fp (FR)	–	M1-Fp (FG)	–
CL-04	M1-Fp (FR)	–	M1-Fp (FG)	–
CL-05	M1-Re (FR)	–	M1-Re (FG)	–
CL-06	M1-Fp (FR)	–	M1-Fp (FG)	–
CL-21	M1-RW (FR)	–	M1-RW (FG)	–
CL-22	M1-RW (FR)	–	M1-RW (FG)	–
CL-23	M1-RW (FR)	–	M1-RW (FG)	–

*Anterograde tracers: BDA, biotinylated dextran amine; FR, FluoroRuby*.

*Retrograde tracers: FG, FluoroGold; TB, True Blue Chloride*.

Following tracer injections, the skin was sutured and treated with antibiotic ointment. Each animal received additional doses of atropine, dexamethasone, and enrofloxacin. Animals were returned to single housed cages for a 7–10 day survival period to allow for tracer transport.

### Histology

Rats were deeply anesthetized with IM injections of ketamine (80 mg/kg) and xylazine (18 mg/kg) and perfused transcardially with heparinized saline, 4% paraformaldehyde, and 4% paraformaldehyde with 10% sucrose. Brains were removed and stored in 4% paraformaldehyde and 30% sucrose at 4°C until saturated.

All brains were sectioned bilaterally into 60-μm slices using a freezing microtome with a slit in the left hemisphere (ventral to the rhinal fissure) to allow proper orientation when mounting. Serially-ordered sections were divided into three series. The first series was mounted on gelatin-coated slides, and then dried and stained with thionin acetate to reveal cytoarchitecture. The second series was processed to visualize BDA using a heavy metal enhanced horse radish peroxidase immunohistochemical reaction as previously described (Kincaid and Wilson, 1996; Smith et al., 2012a). Briefly, sections were first washed in 0.3% H_2_O_2_ to degrade endogenous enzyme activity, rinsed in two 0.3% Triton-X-100 (TX-100) washes, and then incubated for 2 h in avidin-biotin horse radish peroxidase solution mixed in 0.3% TX-100. Sections were then washed twice in 0.1 M PBS and incubated in 0.05% DAB, 0.0005% H_2_O_2_, 0.05% NiCl_2_, and 0.02% CoCl_2_ in 0.1 M tris buffer (pH = 7.2) for 10 min. Two subsequent washes in 0.1 M PBS stopped the reaction. Following immunohistochemistry to visualize BDA, sections were mounted on gelatin-coated slides, dried overnight, dehydrated in ethanol, cleared in xylene and coverslipped with Cytoseal. The third series was directly mounted, dried, dehydrated, defatted, and coverslipped to visualize fluorescent tracers alone.

### Anatomical analysis

All tissue was inspected with an Olympus BH-2 microscope equipped for both brightfield and fluorescent microscopy. Terminals labeled with BDA were visualized with brightfield, whereas TB and FG labeling were visualized with a near UV filter (11000v2; Chroma Technologies), and a TRITC filter (41002, Chroma Technologies) was used for FR labeling. Labeled soma and terminal synapses were plotted and digitally reconstructed using optical transducers attached to the microscope stage (MDPlot, Accustage). For anterograde tracers, beaded varicosities on the axonal terminals were plotted because they represent en passant synapses (Voight et al., 1993; Kincaid and Wilson, 1996). For retrograde tracers, only labeled cells with dendrites were plotted. Digital photomicrographs of brightfield and fluorescent labeling were acquired with a Retiga EX CCD digital camera mounted on the BH-2 microscope. Additional images were obtained with an Olympus FV1000 laser scanning confocal microscope using a 60× oil immersion objective. For TB (405 nm excitation, 410–460 nm emission) and FG (405 nm, excitation, 520–600 nm), sections were scanned sequentially to demonstrate both single and double labeled neurons and were then merged to produce a composite image.

Quantitative analysis of tracer reconstructions was performed using MDPlot software (version 5.1; Accustage). Analysis of the claustrum was confined to sections that contained the striatum because more rostral levels do not contain the claustrum-associated Gng2 protein (Mathur et al., 2009). After the sections were plotted, a grid of 50 μm^2^ bins was superimposed on the reconstructions. Bins containing at least four labeled terminals and one labeled neuron were classified as containing overlapping tracer labeling. Analyses of BDA-FG and BDA-TB overlap were performed separately. The number of overlapping bins was expressed as a percentage of the total number of bins that contained tracer labeling. Statistical analysis was performed using Origin software (version 8.0; Origin Lab). Because BDA processing diminishes the intensity of fluorescence, the third series, which was processed for fluorescence but not BDA, was used to count FG- and TB-labeled and double-labeled neurons.

In addition to our own neuroanatomical tracing experiments, corticoclaustral connectivity in mice was analyzed by accessing data in the Allen Mouse Brain Connectivity Atlas. The analyzed cases were chosen based on the Allen Brain Institute's designation of cortical injection site area. We chose homologous cytoarchitectonic regions and confirmed the functional representation of these regions based on labeling patterns in subcortical structures (see Results).

## Results

To compare the claustral connections with FEF and V1, three rats received different anterograde and retrograde tracers in FEF and V1 of the left and right hemispheres, respectively, (Table 1). Combining different tracer injections in the same animal allowed us to quantify tracer overlap in the claustrum bilaterally and determine the relative strength of corticoclaustral and claustrocortical connections with FEF and visual cortex.

In the first rat, a combined solution of FG and BDA was iontophoretically injected into FEF of the right hemisphere, whereas FR and TB were separately injected into V1 of the left and right hemispheres, respectively. In the other two rats, BDA and FR were separately injected into respective sites in FEF and V1 of the left hemisphere, whereas FG and TB were separately injected into respective sites in FEF and V1 of the right hemisphere.

### Projections from FEF

**Figure 1:**
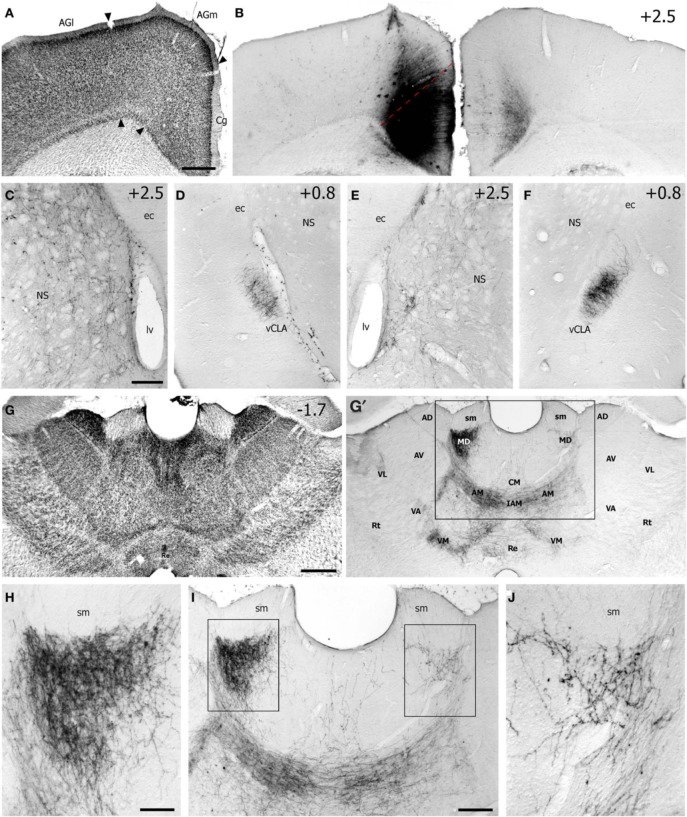
**Case TI-16 demonstrates that the FEF projects to claustrum and other forebrain regions. (A)** Nissl-stained section through the lateral agranular (AGl), medial agranular (AGm), and cingulate (Cg) cortices. **(B)** Deposit of biotinylated dextran amine (BDA) at an M1 site in Cg cortex that evoked eye movements and produced labeled terminals in the contralateral Cg cortex. **(C–F)** The BDA deposit produced labeled terminals in the dorsomedial neostriatum (NS) and ventral claustrum (vCLA) in the left **(C,D)** and right **(E,F)** hemispheres. **(G)** Nissl-stained section of thalamus used to identify BDA-labeled projections **(G′)** in the anterior medial (AM), interanteromedial (IAM), mediodorsal (MD), reuniens (Re), ventromedial (VM), and ventroanterior (VA) nuclei. Box corresponds to **(I)**. **(H–J)** Terminal labeling was densest in the AM and MD nuclei. Boxes in **(I)** indicate **(H,J)**. ec, external capsule; lv, lateral ventricle; sm, stria medularis; AV, anteroventral; AD, anterodorsal; CM, centromedial; Rt, reticular nucleus; VL, ventrolateral. Numbers in **(B–G)** indicate distance from bregma in millimeters. Scale bars: 500 μm in **(A,G)**; 250 μm in **(C,I)**; 100 μm in **(H)**.

**Figure 2:**
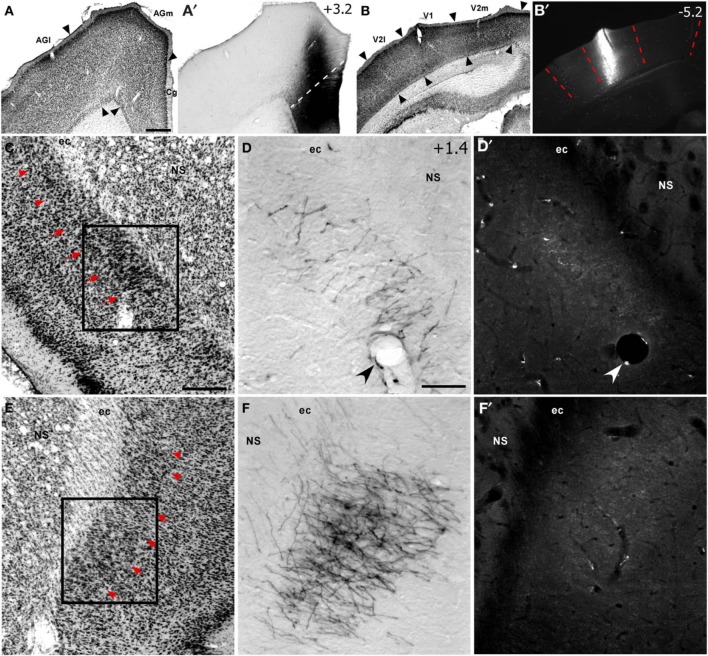
**Corticoclaustral projections from FEF and V1 in case TI-15. (A–B′)** Left hemisphere injections of BDA in FEF **(A,A′)** and FluoroRuby (FR) in V1 **(B,B′)** of the same rat. BDA labeling appeared bilaterally in vCLA **(D,F)**, but was noticeably denser on the contralateral side **(F)**. Sparse FR labeling was apparent only in the ipsilateral vCLA **(D′)**. Boxes in **(C)** and **(E)** correspond to **(D,D′)** and **(F,F′)**, respectively. Red arrowheads demarcate the dorsal claustrum (dCLA) from the vCLA. Black and white arrowheads denote common blood vessels. Scale bars: 500 μm in **(A)**; 250 μm in **(C)**; 100 μm in **(D)**.

In agreement with previous reports (Neafsey et al., 1986; Brecht et al., 2004; Haiss and Schwarz, 2005), cortical sites that evoked eye movements were consistently found at coordinates in the cingulate (Cg) cortex. As shown by Figures 1, 2, tracer injections at these sites were largely confined to CG cortex but some tracer occupied the most medial part of the medial agranular (med-AGm) cortex. While FEF is rostral to M1 sites that evoke rhythmic whisking movements, both FEF and M1-RW reside in Cg and, possibly, the most medial part of AGm (med-AGm) as defined by cytoarchitectonic criteria.

Many BDA-labeled projections from FEF terminated in visual cortex and brainstem regions such as the dorsomedial superior colliculus, periaqueductal gray, oculomotor complex, and the pontine reticular formation. These results corroborate studies that placed rodent FEF in the Cg/med-AGm region on the basis of its connections with oculomotor-related nuclei in the brainstem (Leichnetz et al., 1987; Stuesse and Newman, 1990; Bosco et al., 1994; Guandalini, 2003).

Labeled projections from FEF terminated in the contralateral Cg and other forebrain structures in both hemispheres, including the dorsomedial neostriatum, ventral claustrum (vCLA), and thalamus (see Figure 1). These patterns are similar, but not identical, to projections from the M1 whisker regions, (Alloway et al., 2008, 2009). While projections from FEF terminate more medially in neostriatum than those from M1-Wh, both motor regions project to numerous thalamic regions including the anteromedial (AM), interanteromedial (IAM), paracentral (PC), centrolateral (CL), parafasicular (Pf), reuniens (Re), ventral anterior (VA), and ventromedial (VM) nuclei. The FEF also projects to the ipsilateral mediodorsal (MD) nucleus and, to a lesser extent, to the contralateral MD (Figures 1G′–J), and these projections to MD appear homologous to the FEF projections in primates (Stanton et al., 1988; Sommer and Wurtz, 2004).

Inspection of the claustrum in both hemispheres revealed dense projections from the contralateral FEF. As seen in both Figures 1, 2, BDA injections in FEF produced dense terminal labeling in a large part of the contralateral vCLA, but produced noticeably weaker labeling in a smaller area in the ipsilateral vCLA. These corticoclaustral projections from FEF are remarkably similar to the pattern of corticoclaustral projections that originate from the M1 whisker regions (Smith and Alloway, 2010).

### Projections from V1

We used physiology, cytoarchitecture, and demarcations in the Paxinos and Watson (2007) atlas to confirm the injections in V1. Cytoarchitecturally, V1 is characterized by a prominent granular layer IV, which is not present in surrounding medial and lateral secondary visual cortices, areas 18a and b (Miller and Vogt, 1984b). Substantial amounts of transported tracer in the lateral geniculate nucleus (LGN) of the thalamus further confirmed our injections into V1 (data not shown).

Examination of the claustrum in both hemispheres revealed very sparse projections from V1 cortex. In fact, as shown in Figure 2, the few labeled projections from V1 to the claustrum that were most noticeable were generally located in the ipsilateral hemisphere. By contrast, projections from V1 were observed in several subcortical structures, and many of these overlapped with the projections from FEF. Labeled projections from FEF and V1 overlapped ipsilaterally in the dorsomedial neostriatum, superior colliculus, PC, CL, and lateroposterior (LP) thalamic nuclei. Non-overlapping projections from V1 and FEF appeared in the laterodorsal (LD) thalamus, the dorsal zona incerta (ZI), and in the basal pontine nuclei, in which labeled projections from FEF were observed on both sides of this structure.

### Claustral projections to FEF and V1

**Figure 3:**
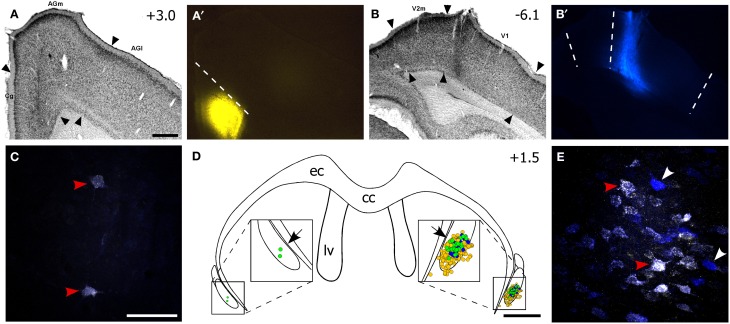
**The ventral claustrum projects to both FEF and V1. (A–B′)** Fluorogold (FG) deposit in FEF and True Blue (TB) in V1 of the right hemisphere for the same case in Figure 2. **(C)** Confocal image of two faintly double-labeled neurons in the left vCLA. **(D)** Reconstruction of labeled soma in the vCLA. Yellow and blue dots are FG- and TB-labeled soma, respectively, and green dots are double-labeled neurons. Black arrows indicate areas in **(C,E)**. **(E)** Confocal image of multiple retrogradely-labeled neurons in the right CLA. Red arrowheads in **(C,E)** indicate double-labeled neurons, white arrowheads indicate TB-labeled neurons. Scale bars: 500 μm in **(A)**; 50 μm in **(C)**; 1 mm in **(D)**.

Injections of FG in FEF and TB in V1 of the same hemisphere produced a dense population of labeled soma in the vCLA of the ipsilateral hemisphere. As shown in Figure 3, FG- and TB-labeled neurons were intermingled in the ipsilateral vCLA, but very few labeled neurons appeared in the contralateral vCLA. A small proportion (7.0 ± 1.2%, mean ± s.e.m.) of the labeled soma were double labeled as shown in confocal images (see Figures 3C,E). Because TB is not easily visualized and is not transported as efficiently as FG, this quantitative measurement of double labeled neurons probably underestimates the proportion of claustral neurons that project to both FEF and V1.

Nonetheless, our plotted reconstructions illustrate partial overlapping populations of FG- and TB-labeled neurons in vCLA. Double-labeled neurons dominated the center of the labeled population (see Figure 3D), and the presence of these neurons indicates that vCLA sends divergent projections to both FEF and V1 in the ipsilateral hemisphere, as reported previously in cats (Minciacchi et al., 1985). This result is similar to our previous observations indicating that the claustrum sends divergent projections to the S1 and M1 whisker regions in the ipsilateral hemisphere (Smith et al., 2012b).

The TB and FG injections also produced intermingled labeled neurons, including double-labeled cells, in several other subcortical regions. Populations of FG- and TB-labeled neurons were intermingled in the ipsilateral intralaminar nuclei (PC, CL, IAM, Pf) and bilaterally in the Re nucleus, which occupies the midline of the thalamus. Prominent labeling, including dual-labeled cells, also appeared in the lateral preoptic area.

### Cortico-claustro-cortical circuit connections

**Figure 4:**
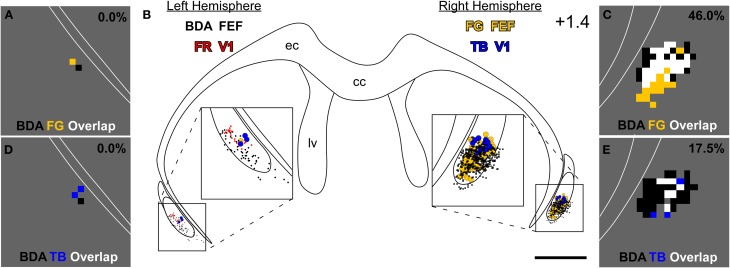
**Projections from the left M1-FEF terminate in the right claustrum, which projects to FEF and V1 in the right hemisphere. (B)** Reconstructions of claustral labeling for the same case depicted in Figures 2, 3. BDA- and FR-labeled terminals shown as black and red dots, respectively; TB- and FG-labeled soma shown as blue- and gold-filled circles, respectively. **(A,C)** Overlap analysis in the left **(A)** and right **(C)** claustrum shows black bins, which contain at least four BDA-labeled varicosities; gold bins, which contain at least one FG-labeled soma; and white bins, which contain both an FG-labeled soma and four BDA-labeled varicosities. **(D,E)** Overlap analysis of BDA-labeled terminals and TB-labeled soma using the same threshold criteria for the bins as in **(A,C)**. Percentages represent fraction of total bins that are colored white in the claustrum of this specific section (i.e., terminal-soma overlap). Scale bars: 1 mm in **(B)**. Bin sizes: 50 μm^2^ in **(A,C–E)**.

Tracer overlap in the claustrum was quantified for the two cases (TI15 and TI16) in which both FEF and V1 were bilaterally injected. In these cases, BDA (FEF) and FR (V1) were deposited on the left side while FG (FEF) and TB (V1) were injected on the right side (Table 1). As shown in Figure 4B, labeling from all four tracers occupied a compact region in the vCLA, spanning no more than 500 μm^2^ within each coronal section. Using 50-μm^2^ bins, a bin had to contain at least four labeled varicosities and one labeled soma to be classified as terminal-soma overlap. This represents the same standard that we used previously to assess cortico-claustro-cortical connectivity (Smith and Alloway, 2010).

Anterograde tracer injections in the left FEF produced dense terminal labeling in the right claustrum. This terminal labeling surrounded the labeled soma produced by retrograde tracer injections in the right FEF. Our overlap analysis indicated that nearly half of the labeled bins in the right claustrum contained both tracers (see Figure 4C). By comparison, terminal-somal overlap in the left claustrum was virtually absent owing to a paucity of labeling from either tracer (Figure 4A). When terminal-soma overlap across all claustral sections was calculated, the proportion of all labeled bins that contained both BDA-labeled terminals and FG-soma (i.e., terminal-somal overlap) was much larger contralateral to the FEF-BDA injection (43.3 ± 4.6%) than ipsilaterally (5.3 ± 4.3%). Hence, the FEF projects mainly to the contralateral claustrum, which then projects to the FEF in that hemisphere to create an interhemispheric cortico-claustro-cortical circuit between the FEF regions in the two hemispheres.

A similar pattern was found for the connections between FEF and the contralateral V1 region. As shown by the section reconstructed in Figures 4D,E, terminal-somal overlap was 17.5% in the claustrum contralateral to the FEF-BDA injection but was 0% in the ipsilateral claustrum. When terminal-somal overlap was calculated for all sections through the claustrum that contained labeled bins, the proportion of bins that contained overlap was larger in the claustrum contralateral to the FEF-BDA injection (37.2 ± 3.0%) than in the ipsilateral claustrum (11.4 ± 1.2%). These findings clearly demonstrate the presence of an interhemispheric cortico-claustro-cortical circuit in which the FEF transmits information disynaptically to the contralateral V1 by means of its projections to the contralateral claustrum.

### Retrograde confirmation of corticoclaustral projections

**Figure 5:**
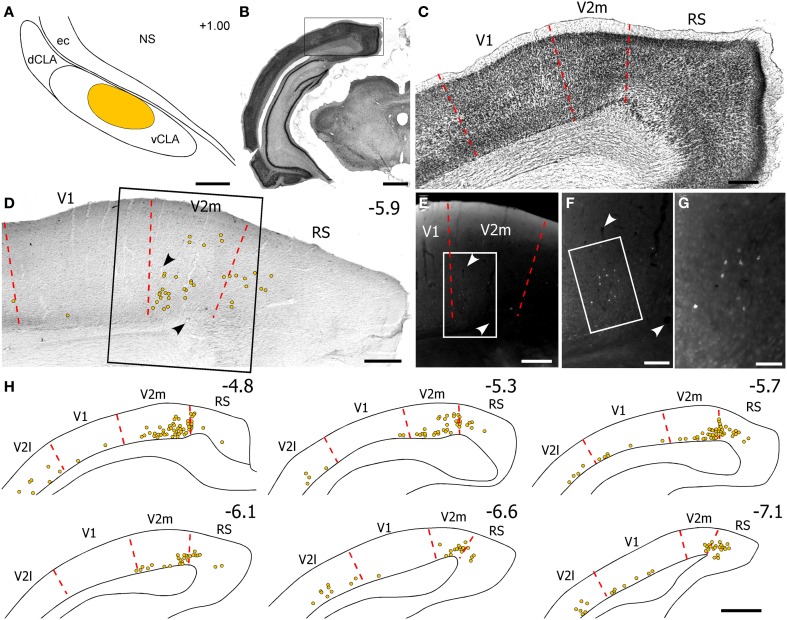
**Few neurons in visual cortex project to the claustrum. (A)** Reconstruction of an FG deposit in claustrum depicted in Figure 9 of Smith and Alloway (2010). **(B,C)** Nissl-stained section through primary visual (V1), medial secondary visual (V2m), and retrosplenial cortices. **(D)** Plotted location of FG-labeled neurons in an adjacent section. Inset in **(D)** indicates location of **(E)**. **(F,G)** Successive magnifications of retrogradely-labeled soma in V2m. **(H)** Digital reconstructions of FG-labeled neurons in visual cortex. Numbers indicate caudal distance from bregma. Scale bars: 250 μm in **(A,C–E)**; 1 mm in **(B,H)**; 100 μm in **(F)**; 50 μm in **(G)**.

Our anterograde tracing results indicate that V1 sends very weak projections to the claustrum, whereas FEF sends dense projections to vCLA. To confirm this finding, we inspected data from our previous study in which we injected FG into the claustrum (see Figure 9 in Smith and Alloway, 2010). In that case, the contralateral frontal cortex contained many retrogradely-labeled neurons in the Cg and medial AGm regions (see Figures 10, 11 in Smith and Alloway, 2010), but no labeled neurons were observed in the S1 barrel region of either hemisphere. In the occipital region, however, separate populations of FG-labeled neurons were found ipsilaterally (data not reported previously). As indicted by Figure 5, a few labeled neurons appeared in layer VI of primary visual cortex (V1) and lateral secondary visual cortex (V2l), but many more labeled neurons were observed in the medial part of the secondary visual cortex (V2m), which is consistent with previous reports (Miller and Vogt, 1984a; Carey and Neal, 1985).

### Functional topography of claustral connections with motor cortex

**Figure 6:**
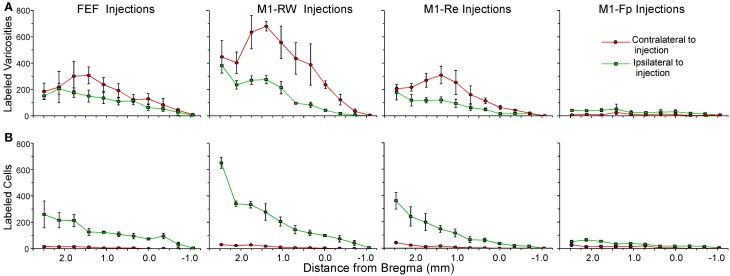
**Distribution of mean terminal and cell counts from tracer injections in different parts of rat motor cortex. (A)** Labeled terminal counts in the ipsilateral and contralateral claustrum from BDA and FR injections in FEF and in the rhythmic whisking (M1-RW), whisker retraction (M1-Re), and forepaw (M1-Fp) regions from a previous study (Smith and Alloway, 2010). **(B)** Labeled cell counts in the claustrum of both hemispheres from FG injections in the same regions. Symbols in each line graph represent the mean counts per section from three tracer injection cases; error bars represent standard error of the mean.

The claustral connections with FEF in the present study were compared to the claustral connections for the M1 whisker (M1-RW, M1-Re) and M1 forepaw (M1-Fp) representations that we characterized previously (Smith and Alloway, 2010). Figure 6 shows the rostrocaudal distribution of claustral labeling produced by injecting retrograde (Figure 6A) or anterograde (Figure 6B) tracers into these four motor regions (summary of injections in Table 1). Statistical analysis revealed significant effects for injection location and hemispheric labeling for both anterograde (Injected area: *F* = 34.2; *p* < 0.00001; Hemispheric labeling: *F* = 25.1; *p* < 0.00001) and retrograde injections (Injected area: *F* = 10.5; *p* < 0.00001; Hemispheric labeling: *F* = 95.1; *p* < 0.00001).

The FEF, M1-RW, and M1-Re regions all project significantly more strongly to the contralateral than to the ipsilateral claustrum (FEF, paired *t* = 2.46, *p* < 0.05; M1-RW, paired *t* = 6.26, *p* < 0.000001; M1-Re, paired *t* = 5.59, *p* < 0.00001). Following retrograde tracer injections into these motor regions, however, the number of labeled neurons is much larger ipsilaterally (FEF, paired *t* = 7.34, *p* < 0.0000001; M1-RW, paired *t* = 6.46, *p* < 0.000001; M1-Re, paired *t* = 5.38, *p* < 0.00001). By comparison, labeling produced by tracer injections in M1-Fp is extremely weak in both directions and is present almost entirely on the ipsilateral side (anterograde labeling, *t* = 6.04, *p* < 0.000001; retrograde labeling, *t* = 5.60, *p* < 0.00001).

**Figure 7:**
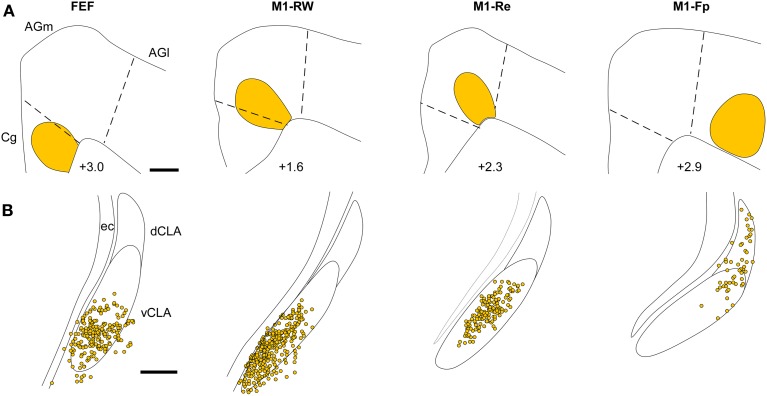
**Representative examples illustrating the topography of labeled neurons in the claustrum produced by tracer injections in FEF, M1-RW, M1-Re, and M1-Fp. (A)** Reconstructions of the M1 tracer injections in the current study (case TI-15) and in cases CL-03, CL-05, and CL-21 illustrated in Figures 1–3 of Smith and Alloway (2010). **(B)** Reconstruction of FG-labeled neurons in the claustrum from each injection in **(A)**. Scale bars: 500 μm in **(A)**; 250 μm in **(B)**.

The retrograde labeling patterns observed in the claustrum in the present study were compared with three cases of retrograde labeling in the claustrum (CL01, CL05, and CL21) that were illustrated previously in Figures 1–3 of Smith and Alloway (2010). These comparisons indicate that the claustrum has a distinct functional topography. As shown in Figure 7, each ICMS-defined and tracer-injected motor region is linked to a specific part of the claustrum. The FG injections in FEF (case TI-15) and in M1-RW (case CL21), which occupy the Cg/med-AGm region, produced retrograde labeling in the deepest parts of the vCLA. The FG injection in M1-Re (case CL05), which is centered in AGm, produced labeling in the middle of vCLA. Finally, an FG injection in M1-Fp (case CL03), which occupies AGl, revealed labeled neurons mainly in the dCLA. These data indicate that the claustrum has a topographic organization in which the medial to lateral extent of M1 cortex is represented ventral to dorsal in the claustrum.

These claustrum subdivisions are defined not only by the specificity of their inputs from motor cortex, but also by their projections to different sensory regions. Comparison of the retrograde labeling in the present study with those from our previous report (Smith et al., 2012b), indicates that vCLA projects to both FEF and V1, whereas the middle of the claustrum projects to both M1-Re and S1-Wh.

### Corticoclaustral projections in mice

We inspected the corticoclaustral connections in the Allen Mouse Brain Connectivity Atlas (2012), and focused on cases with tracer injections in S1, V1, Cg, AGm, and AGl. We examined mouse cases in which the tracers filled all cortical layers and the injection locations appeared equivalent to our injection sites as determined by the surrounding anatomical landmarks. Finally, we analyzed whether the terminal labeling patterns in the forebrain and brainstem matched the patterns seen in our rat experiments to assure functional homology with our data. We observed, for example, that Cg injections in mice produced labeling in the dorsomedial neostriatum, nucleus MD in the thalamus, dorsomedial superior colliculus, and the ocular motor complex in the midbrain. This pattern of labeling is completely consistent with the patterns that we observed when anterograde tracers were deposited in the FEF (Cg cortex) of rats. Likewise, tracer injections in AGm or AGl of mice produced subcortical labeling patterns that are consistent with our anterograde tracer injections at sites where ICMS evoked movements of the whiskers or forelimb (Alloway et al., 2008, 2009, 2010).

**Figure 8:**
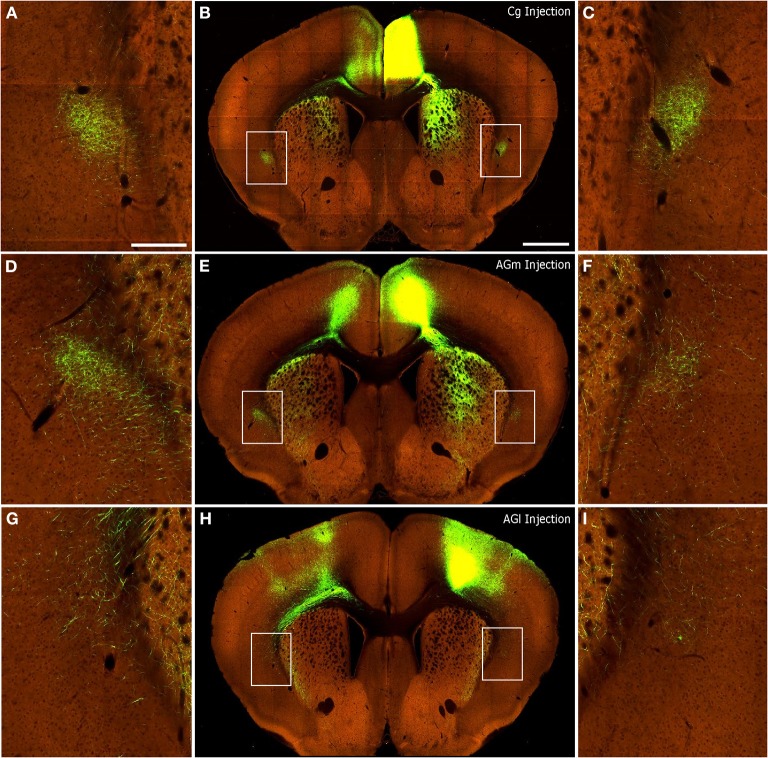
**Corticoclaustral projections from Cg, AGm, and AGl in mice**. Images of AAV injections and subsequent labeling acquired from the Allen Mouse Brain Connectivity Atlas (2012). Center panels show images of labeling from representative AAV tracer injections in Cg **(B)**, AGm **(E)**, and AGl **(H)**. Hyperlinks connect to the complete data sets on the Allen Institute website. In each case, labeling appears in the contralateral cortex as well as bilaterally in the striatum and claustrum. **(A,D,G)** correspond to insets of the claustrum in the left hemisphere of center panels. **(C,F,I)** likewise correspond to insets of the claustrum in the right hemisphere of center panels. Scale bars: 250 μm in **(A)**; 1 mm in **(B)**.

Qualitative inspection of the injections in the S1-Wh (Experiment#: 126908007, 127866392) region matched our previous finding that rat S1 does not project to the claustrum (Smith et al., 2012b). Mice that received injections in V1 showed sparse labeling in the ipsilateral claustrum (Experiment#: 113887162, 100141599), which corresponds to our findings when rat V1 region is injected. Finally, as shown in Figure 8, injections into Cg (Figures 8A–C; Experiment# 112514202), AGm (Figures 8D–F; Experiment# 141603190), and AGl (Figures 8G–I, Experiment# 141602484) display patterns of interhemispheric corticoclaustral labeling that are highly similar to our results in the rat. In mice, as in rats, the majority of coronal sections containing the claustrum indicate that the AGm and Cg regions project more strongly to the contralateral than to the ipsilateral claustrum (confirming findings by Mao et al., 2011), whereas AGl has sparse connections with the claustrum in either hemisphere.

## Discussion

By placing different anterograde and retrograde tracers in FEF and V1 of both hemispheres, this study revealed several new findings about the functional organization of the rat claustrum. Most significantly, rat FEF sends dense projections to the contralateral claustrum, but sends relatively weak projections to the ipsilateral claustrum. The claustrum receives weak projections from ipsilateral V1 but is not innervated by the contralateral V1. When different retrograde tracers are injected into FEF and V1 of the same hemisphere, many intermingled and double-labeled neurons appear in the ipsilateral, but not the contralateral, claustrum.

These results indicate that the claustrum is part of an interhemispheric circuit for transmitting information from FEF to separate visuomotor regions in the other hemisphere. Our previous work shows that the claustrum has a parallel set of circuit connections with the M1 and S1 whisker regions (Smith and Alloway, 2010; Smith et al., 2012b). Collectively, these findings indicate that the claustrum has a role in the interhemispheric transmission of certain types of sensorimotor information.

**Figure 9:**
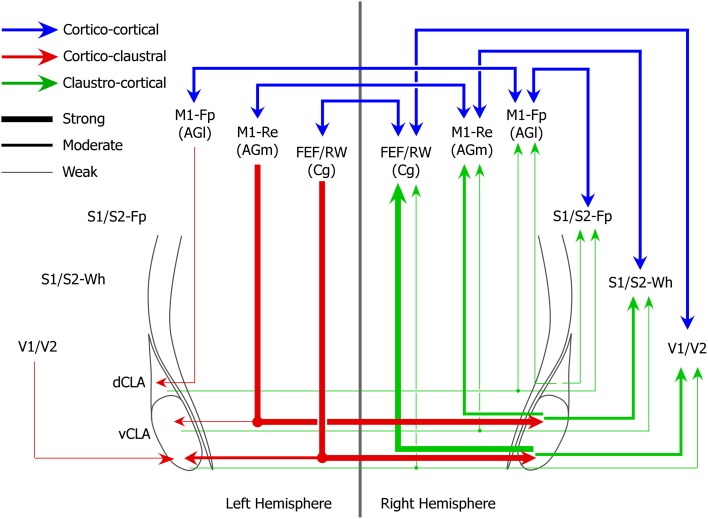
**Circuit diagram of interhemispheric sensorimotor cortico-claustro-cortical circuits in rats**. Strength of projections are indicated by line thicknesses (see legend).

While the claustrum receives dense interhemispheric projections from cortical motor regions that regulate movements of the whiskers and eyes, the M1 limb regions send very weak projections to the claustrum and only within the same hemisphere. These differences in the density of corticoclaustral projections from different parts of M1 are also apparent in the Allen Mouse Brain Connectivity Atlas. A summary of these functional differences in claustral connectivity is illustrated in Figure 9.

Our last major finding is that the claustrum has a well-defined functional topography along its dorsoventral axis. The M1 forepaw region projects to the dorsal claustrum, the M1 whisker region projects to the middle claustrum, and the FEF region projects to the vCLA. Likewise, the S1 forelimb, the S1 whisker, and the V1 regions receive projections from the dorsal, middle, and vCLA, respectively.

### Visuomotor claustrum circuitry

Corticoclaustral projections from the frontal and occipital cortices differ both qualitatively and quantitatively. The FEF projects densely to the contralateral claustrum, but only weakly to the ipsilateral claustrum. Visual cortex sends some projections to the ipsilateral claustrum, but these originate mainly from V2m, which also projects to FEF and the ventral superior colliculus, regions known for controlling saccadic eye movements (Wang and Burkhalter, 2013; Wang et al., 2013). The corticoclaustral projections from both FEF and V2m originate from layer V, which is significant because this layer contains corticobulbar motor output neurons. These facts indicate that rat vCLA has a role in processing information concerned with eye movements.

The lack of reciprocal projections between certain cortical areas and the claustrum provides some clues about the function of the claustrum. While FEF projects strongly to the contralateral claustrum, the claustrum projects ipsilaterally to FEF but does not send feedback projections to the contralateral FEF. Likewise, the connections between the claustrum and primary visual cortex are not reciprocal. The claustrum projects strongly to ipsilateral V1, but reciprocal projections from V1 to the claustrum are practically nil (Miller and Vogt, 1984a; Carey and Neal, 1985). After placing different retrograde tracers in FEF and V1 of the same hemisphere, we observed many double-labeled neurons in the ventral part of the ipsilateral claustrum, and this indicates that identical information is transmitted from vCLA to both FEF and V1.

When these divergent claustral projections to V1 and FEF are considered with the relative weakness of corticoclaustral feedback projections in the same hemisphere, the emerging circuit suggests that the claustrum is important for coordinating V1 and FEF processing in the same hemisphere.

### Functional topography in the claustrum

Our studies demonstrate that rat claustrum has a well-defined functional topography. In a previous report we showed that the M1 forelimb region is linked to dCLA, whereas the M1 whisker region is connected to vCLA (Smith and Alloway, 2010). The present study extends this work by showing that visuomotor cortical areas are connected to the most ventral part of vCLA. We have observed intraclaustral connections along the rostrocaudal, but not the dorsoventral axis (Smith and Alloway, 2010). This anisotropic organization of intraclaustral connectivity is consistent with the segregation of unimodal responses in different subregions of the primate claustrum (Remedios et al., 2010).

### Theoretical function of interhemispheric sensorimotor claustral circuits

We recently injected different retrograde tracers into S1 and M1, and we observed many double-labeled neurons in the claustrum (Smith et al., 2012b). In the present study we observed many double-labeled claustral neurons after injecting different retrograde tracers into FEF and V1. These results are consistent with Type A and B claustral neurons that were previously reported in the brains of rats and cats (Minciacchi et al., 1985). Selective placement of different retrograde tracers in cortex of the same animals has revealed claustral neurons that innervate both ipsilateral and contralateral frontal regions (Type C neurons), and others that innervate the contralateral frontal and ipsilateral occipital regions (Type D neurons). Other studies on a variety of mammalian species have identified specific claustral regions that project to sensory and motor cortical areas, including divergent projections to the S1 and S2 cortices (Li et al., 1986; Sadowski et al., 1997; Jakubowaska-Sadowska et al., 1998).

The presence of double-labeled neurons demonstrates that the claustrum conveys the same information to separate, but functionally-related cortical areas. While the exact nature of the information that is transmitted to FEF and V1 (or to the M1 and S1 whisker regions) remains unknown, claustral divergence provides a mechanism for ensuring simultaneous processing of the same information in separate cortical regions.

Our findings in two different sensorimotor systems indicate that dense interhemispheric projections to the claustrum originate from motor regions in frontal cortex. Mounting evidence indicates that these frontal regions (Cg, AGm) in the rat are involved not only in motor control, but also in directed-attention and memory-guided orienting behaviors (Reep and Corwin, 2009; Erlich et al., 2011; Boly et al., 2013).

Transmission of attention-related motor signals to the claustrum is supported by the fact that the several intralaminar thalamic nuclei also have connections with the claustrum. Many tracing studies have reported that the claustrum receives non-reciprocal projections from the centromedial, CL, PC, and Pf nuclei (Kaufman and Rosenquist, 1985; Sloniewski et al., 1986b; Vertes et al., 2012; Alloway et al., 2014). Substantial evidence implicates these intralaminar nuclei with a critical role in attention and conscious perception, (Hudetz, 2012), and these connections suggest that the claustrum is involved in dispersing attention-dependent signals during the conscious state.

Consistent with our past work (Smith and Alloway, 2010; Smith et al., 2012b), the present study supports our hypothesis that claustral connections enable interhemispheric transmission of certain types of modality-specific information to widely-separated cortical areas. By transmitting information from the frontal cortex in one hemisphere to parietal and occipital regions in the other hemisphere, the claustrum provides an interhemispheric route that extends beyond the other callosal projections that interconnect corresponding sites in both hemispheres.

Ocular saccades and whisking are rapid movements involved in the active acquisition of visual and somesthetic information from both sides of the body. These movements are purposeful, they require a conscious state, and they are dynamically modulated by sensory inputs. In addition to callosal connections between corresponding cortical areas in the two hemispheres, the claustrum provides a node for transmitting attention-dependent sensorimotor signals from one frontal region to multiple sensorimotor regions in the other hemisphere. This is especially relevant when attention is directed toward improving the acquisition and interpretation of sensory inputs that may come from a broad expanse of extra-personal space. Indeed, studies in the human visual system have indicated that callosal connections and subcortical circuits are involved in interhemispheric visuomotor integration, “promoting a unified experience of the way we perceive the visual world and prepare our actions” (Schulte and Muller-Oehring, 2010). In our view, the claustrum facilitates interhemispheric corticocortical transmission so that multiple sensorimotor cortical regions can work together to produce a stable global percept out of the rapidly shifting sensory information coming in from sensors on both sides of the head.

### Conflict of interest statement

The authors declare that the research was conducted in the absence of any commercial or financial relationships that could be construed as a potential conflict of interest.
